# BMP‐2 induces human mononuclear cell chemotaxis and adhesion and modulates monocyte‐to‐macrophage differentiation

**DOI:** 10.1111/jcmm.13814

**Published:** 2018-08-13

**Authors:** Evangelia Pardali, Lena‐Maria Makowski, Merle Leffers, Andreas Borgscheiper, Johannes Waltenberger

**Affiliations:** ^1^ Department of Cardiovascular Medicine University Hospital of Münster Münster Germany; ^2^ Cells‐in‐Motion Cluster of Excellence (EXC 1003 ‐ CiM) University of Münster Münster Germany

**Keywords:** adhesion, atherosclerosis, BMP, chemotaxis, diabetes, endothelial cells, monocytes

## Abstract

Type 2 diabetes mellitus (T2DM) is a cardiovascular risk factor which leads to atherosclerosis, an inflammatory disease characterized by the infiltration of mononuclear cells in the vessel. Bone morphogenetic protein (BMP)‐2 is a cytokine which has been recently shown to be elevated in atherosclerosis and T2DM and to contribute to vascular inflammation. However, the role of BMP‐2 in the regulation of mononuclear cell function remains to be established. Herein, we demonstrate that BMP‐2 induced human monocyte chemotaxis via phosphoinositide 3 kinase and mitogen‐activated protein kinases. Inhibition of endogenous BMP‐2 signalling, by Noggin or a BMP receptor inhibitor, interfered with monocyte migration. Although BMP‐2 expression was increased in monocytes from T2DM patients, it could still stimulate their migration. Furthermore, BMP‐2 interfered with their differentiation into M2 macrophages. Finally, BMP‐2 both induced the adhesion of monocytes to fibronectin and endothelial cells (ECs), and promoted the adhesive properties of ECs, by increasing expression of adhesion and pro‐inflammatory molecules. Our data demonstrate that BMP‐2 could exert its pro‐inflammatory effects by inducing monocyte migration and adhesiveness to ECs and by interfering with the monocyte differentiation into M2 macrophages. Our findings provide novel insights into the mechanisms by which BMP‐2 may contribute to the development of atherosclerosis.

## 1 INTRODUCTION

Atherosclerosis is characterized by increased inflammation induced by systemic and local factors, which leads to endothelial cell (EC) inflammation, hyperproliferation of smooth muscle cells (SMCs), recruitment of mononuclear cells, their differentiation into macrophages and the development of atherosclerotic lesions.^1^ In addition, it has been shown that calcium deposits (vascular calcification) influence atherosclerotic plaque stability and possibly the incidence of acute coronary syndrome.^2^ Although EC dysfunction is a critical step in the onset of atherosclerosis, monocytes play an important role in the development of atherosclerosis. Cardiovascular risk factors such as type 2 diabetes mellitus (T2DM) accelerate monocyte recruitment, adhesion on the endothelium and their infiltration into the vessel wall. There monocytes start to proliferate, differentiate to macrophages and finally into foam cells leading to increased local inflammation and plaque development.^1^, ^3^ Elevated numbers and increased recruitment of monocytes have been linked to atherosclerotic plaque formation, while inhibition of monocytes leads to decreased size of atherosclerotic plaques.^1^, ^3^ Additionally, monocytes play an important role in the formation of new collaterals as they are recruited to sites of arteriogenesis by cytokines such as vascular endothelial growth factor A (VEGFA). Our previous studies demonstrated that T2DM results in impaired VEGFA‐induced monocyte migratory responses and it has been suggested that this may contribute to the decreased formation of collateral vessels in patients with T2DM.^3^, ^4^

Bone morphogenetic proteins (BMPs) are members of the transforming growth factor (TGF)‐β superfamily and play a crucial role in development, cell differentiation, bone formation and vascular function.^5^, ^6^ BMPs signal through transmembrane heteromeric complexes of type I and II serine‐threonine kinase receptors.^5^, ^6^ BMP binding induces constitutively active type II receptors to transphosphorylate and activate type I receptors, which in turn phosphorylate the intracellular receptor‐associated (R)‐Smads Smad1/5/8. R‐Smads form then complexes with the common Smad4 and translocate into the nucleus where they regulate transcription of target genes.^5^, ^6^ Besides the Smad‐mediated signalling pathway BMPs activate also additional pathways including mitogen‐activated protein kinases (MAPKs) p38, phosphoinositide 3‐kinase (PI3K) and others. BMP signalling can be modulated by extracellular ligand antagonists, such as noggin, chordin or cerberous.^5^ Several studies have suggested that BMPs are involved in vascular inflammation, vessel calcification and atherosclerosis.^7^, ^8^, ^9^, ^10^ BMP‐2 is expressed in calcified atherosclerotic plaques^11^, ^12^ and in calcified arteries in mouse models.^13^, ^14^ Moreover, increased BMP‐2 plasma levels were associated with atherosclerosis and coronary calcification in T2DM patients.^15^ In addition, BMP‐2 can induce EC activation^10^ and promote calcification of ECs, myofibroblasts and SMCs.^16^, ^17^ Furthermore, interference with the BMP‐2 pathway hinders atherosclerosis, while enhanced BMP‐2 activity results in increased atherosclerotic lesion formation in mice.^9^, ^10^, ^18^ It was suggested that the effects of BMP‐2 on atherosclerosis development are due to the effects of BMP‐2 signalling on EC function. Whether the effects of BMP‐2 on atherosclerosis development are also due to its effects on monocyte function is not known.

This study focuses on the characterization of the effects of BMP‐2 on mononuclear cell function, as well as their interaction with ECs and addresses the mechanisms involved therein. To this extent, we used human peripheral blood monocytes and demonstrated that BMP‐2 expression is increased in monocytes from T2DM patients. BMP‐2 is a potent monocyte chemoattractant and interferes with monocyte differentiation into M2 macrophages. Moreover, we demonstrate that induces ECs‐mononuclear cell interactions. Altogether our results provide novel insights into the mechanisms by which BMP‐2 exerts its pro‐inflammatory effects and in this way may contribute to the development of atherosclerosis.

## 2 MATERIAL AND METHODS

### 2.1 Cells and reagents

Cell culture media RPMI 1640 Medium GlutaMAX™, DMEM and M199 were obtained from Life technologies. Recombinant human BMP‐2 and Tumour necrosis factor‐α (TNF‐α), M‐CSF, IL‐1β and IL‐4 were obtained from Peprotech Inc., (Rocky Hill, CT, USA) and human VEGFA from Reliatech. PI3K inhibitor Wortmannin, p38 inhibitor SB239063, and ERK1/2 kinase inhibitor PD98059 and the BMP kinase receptor inhibitor LDN193189 were purchased from Sigma‐Aldrich (Saint Louis, MO, USA). Antibodies recognizing phosphorylated forms of SMAD1 (Ser456/467), AKT (Ser473), ERK1/2 (Thr202/Tyr204), and p38 (Thr180/Tyr182) and total AKT and ERK1/2 were purchased from Cell Signaling Technology Inc., (Danvers, MA, USA). The total p38 and Smad1/5/8 antibodies were obtained from SantaCruz. β‐Actin antibody was obtained from Sigma‐Aldrich.

### 2.2 Characterization of patients

This study conforms to the principles of the Declaration of Helsinki, and it was approved by the scientific and ethics committee of the University of Münster. Written informed consent was obtained from all subjects. Patients (a) without type 2 diabetes mellitus (nT2DM) and (b) with type 2 diabetes mellitus (T2DM) were recruited during a routinely check up in the Department of Cardiology at the University Hospital Münster. Clinical parameters of the study population are presented in Table 1. Monocytes were also isolated from human blood leucocyte reduction chambers from healthy individuals recruited by the blood bank of the University Hospital Münster.

**Table 1:** Clinical baseline characteristics of the study population

	nT2DM (n = 36)	T2DM (n = 13)
Age (y)	58.39 ± 9.26	60.38 ± 6.33
Gender (male/female)	23/13	9/4
CAD (no/yes)	19/17	3/10
Stenosed vessel	0.97 ± 1.25	1.75 ± 1.29
Hypertension (no/yes)	12/24	1/12
Smoker (no/yes)	17/19	8/5
Dyslipidaemia (no/yes)	22/14	7/6
Obesity (no/yes)	30/6	6/7
Glucose (mg/dL)	103.50 ± 13.53	158.08 ± 35.8
HbA1c (%)	5.87 ± 0.39	7.44 ± 0.79
Cholesterol (mg/dL)	181.22 ± 40.77	180.23 ± 41.2
LDL cholesterol (mg/dL)	118.2 ± 28.3	100.24 ± 37.36
hsCRP (mg/dL)	0.25 ± 0.25	0.4 ± 0.46
Leucocytes (ths/μL)	6.34 ± 1.91	7.5 ± 2.26
Monocytes (ths/μL)	0.93 ± 2.66	0.53 ± 0.13

T2DM, type 2 diabetes mellitus.

The values are presented as mean ± standard deviation (SD) unless it is indicated differently.

### 2.3 Human primary monocyte isolation

Monocytes were isolated as described previously^19^, ^20^ Monocytes were isolated by means of density gradient centrifugation, followed by a magnetic‐bead‐based immunological isolation assay. In brief, density centrifugation was performed with Histopaque (1.077 g/mL) separation (Sigma‐Aldrich Inc.) to isolate peripheral blood mononuclear cells. Subsequently, monocytes were extracted from the mononuclear fraction using the MACS monocyte isolation kit II (Miltenyi Biotec GmbH, Bergische Gladbach, Germany), according to the manufacturer's protocol.

### 2.4 Monocyte chemotaxis

Chemotaxis assays were performed with Boyden chamber methodology as previously described^19^, ^20^ using a 48‐well Boyden chamber (Neuroprobe) and Nucleopore PET membrane (Whatman) with 5‐μm‐diameter pores. Cells in a concentration from 0.5 × 10^6^ cells/mL were allowed to migrate for 90 minutes at 37°C and 5% CO_2_. Only the cells migrated through the pores were counted. In the experiments where pharmacological inhibitors were used, the cells were preincubated with the inhibitors for 30 minutes prior to the chemotaxis/chemokinesis experiments.

### 2.5 Macrophage differentiation

Freshly isolated monocytes were cultured in RPMI medium (containing 10% FBS and 1.25% Penicillin/Streptomycin). Monocyte differentiation into macrophages was induced by addition of 50 ng/mL M‐CSF. Differentiation into M1 or M2 macrophages was induced by addition of 10 ng/mL IL‐1β or 10 ng/ml IL‐4, respectively, after 7 days of differentiation.

### 2.6 Protein analysis

Monocytes or endothelial cells were stimulated with different concentrations of the indicated ligands and for the indicated lengths of time. Thereafter, cells were processed to lysates and subjected to Western blot analysis as described previously.^19^

### 2.7 Adhesion assay of mononuclear cells to Fibronectin

Forty‐eight‐well plates were coated with 10 μg/mL fibronectin (Sigma‐Aldrich) overnight at 37°C. Unspecific binding sites were blocked with 1% BSA in PBS at least 1 hour at 37°C. Besides, 1% BSA coated wells served as negative control. Primary monocytes were stimulated with and without 50 ng/mL BMP‐2 for 60 minutes at 37°C. Afterwards, cells were plated (1 x 10^5^ cells/well) and allowed to adhere for 15 minutes at 37°C. Subsequently, nonadhered cells were washed away by swinging gently 2‐5 times with prewarmed PBS. Pictures were taken of the well‐middle, and the cells were counted.

### 2.8 Adhesion assay of mononuclear cells to endothelial cells

Human umbilical vein endothelial cells (HUVECs) were isolated from anonymized donors according to the Declaration of Helsinki and approved by the ethics boards of the University of Münster (2009‐537‐f‐S). Human umbilical vein endothelial cells were cultivated as described before.^20^ HUVECs from different donors were seeded on 48‐well plates and cells were allowed to grow for 2 days until they reached confluence. Confluent HUVECs were stimulated with the ligands in M199 medium containing 2% FBS for 5 hours at 37°C. Mononuclear cells were labelled with 4 pg/mL calcein for 15 minutes and washed once with warm medium prior to the adhesion assy. After incubation, HUVECs were washed, and the calcein‐labelled mononuclear cells (2 x 10^5^ cells/well) were added for 20 minutes. Nonadherent cells were removed by washing 3‐4 times with PBS and pictures were taken with a fluorescent Leica microscope. The adherent cells were counted using ImageJ. Alternatively, human monocytes were stimulated with BMP‐2 for 30 minutes, labelled with 4 pg/mL calcein for 15 minutes, washed once with warm medium and added (2 x 10^5^ cells/well) to confluent HUVEC monolayers for 20 minutes. Nonadherent cells were removed by washing 3‐4 times with PBS and pictures were taken with a fluorescent Leica microscope. The adherent cells were counted using ImageJ.

Mouse brain endothelial cells bEnd5 were grown in DMEM (high glucose, 4.5 g/L), supplemented with 10% FBS, 100 U/mL penicillin and 100 g/mL streptomycin, on plates coated with 1% gelatin in a humidified 37°C incubator with 5% CO_2_. For adhesion assays using mouse bEnd5 endothelial cell line, the cells were seeded on 48‐well plates and allowed to grow for 2 days until they reached confluence. They were stimulated overnight with the ligands, prior to the addition of labelled mononuclear cells. In some experiments, endothelial cells were preincubated for 30 minutes in the presence or absence of inhibitors dissolved in DMSO and then the ligands or medium only (control) were added. Then, the adhesion assay was performed as described above.

### 2.9 PCR expression analysis

Total RNA was extracted using the NucleoSpin RNAkit (Macherery‐Nagel) and first strand cDNA synthesis and real‐time PCR was performed as previously described.^21^ Gene expression levels were determined with the comparative Δ*C*t method using 18sRNA as reference. Sequences of the primers used for cDNA amplification in the quantitative RT‐PCR experiments are detailed in Tables S1 and S2.

### 2.10 Statistical analysis

The data are presented as mean ± SEM using GraphPad Prism. Data were analysed using the nonparametric Kruskal‐Wallis test with Dunn for the comparison of more than two groups. For the comparison of two groups with parametric distribution of the data the *t* test and for nonparametric distributed data the unpaired *t* test or Wilcoxon signed‐rank test was used. The generalized linear mixed model (GLMM) was used for the analysis of the results from the migration assays with monocytes from patients. A probability (*P*‐) value of <0.05 was considered statistically significant. *P* values: *P < 0.05, **P < 0.01, ***P < 0.001.

## 3 RESULTS

### 3.1 BMP‐2 induces mononuclear cell chemotaxis

**Figure 1:**
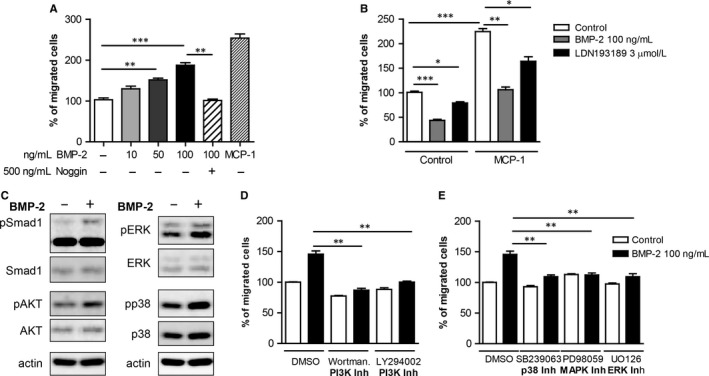
BMP‐2 induces monocyte migratory responses in a dose‐dependent manner via induction of PI3K, p38 and ERK signalling cascades. A, Monocytes were characterized for their chemotactic activity towards different concentrations of BMP‐2 and MCP‐1 (10 ng/mL). Noggin, a BMP‐2/4/7 inhibitor, inhibited the effect of BMP‐2 on monocyte migration (n = 5). B, Monocytes were treated with 500 ng/mL noggin or with 3 μmol/L LDN193189 for 30 min, and they were characterized for their basal and MCP‐1‐induced (10 ng/mL) chemotactic activity. (n = 5). C, Monocytes were stimulated with 100 ng/mL BMP‐2 for 10 min. Phosphorylation of Smad1, p38, ERK1/2 and AKT was determined by Western blot. D, Monocytes were incubated for 30 min with vehicle or different PI3K kinase inhibitors: 25 nmol/L of Wortmannin or 1 μmol/L LY29400 and they were assessed for their migratory responses towards 100 ng/mL BMP‐2 (n = 5). E, Monocytes were incubated for 30 min with vehicle or different MAPK kinase inhibitors, 10 μmol/L SB239063, 25 μmol/L PD98059 or 10 μmol/L U0126 and characterized for their migratory responses towards 100 ng/mL BMP‐2 (n = 5). Data are presented as mean ± SEM (**P < 0.01, ***P < 0.001)

BMP‐2 can induce migration of various cells.^22^ To analyse the effects of BMP‐2 on mononuclear cell movement, we performed chemotaxis assays. Our results demonstrated that BMP‐2 induces monocyte chemotaxis in a dose‐dependent manner (Figure 1A). The effects of BMP‐2 were neutralized by addition of noggin, a natural antagonist of BMP‐2/4/7^5^, which binds to BMPs and inhibits the binding to their receptors. In conclusion, our results suggest that BMP‐2 acts as a potent monocyte chemoattractant.

To characterize the role of endogenous BMP‐2/4 signalling on mononuclear cell migration, monocytes were treated with noggin or the BMP type I receptors (ALK2 and ALK3) LDN193189 prior to the migration assay (Figure 1B). Interestingly, noggin pretreatment of monocytes resulted in inhibition by 60% of the basal mononuclear migratory responses. In addition, noggin inhibited the monocyte chemoattractant protein (MCP)‐1‐induced migration by 50% reduction. Monocyte chemokinesis and MCP‐1‐induced monocyte migration were reduced by 20%‐25% when monocytes were pretreated with LDN193189. These results suggest that endogenous BMP‐2 signalling plays an important role in monocyte chemokinesis and chemotaxis.

### 3.2 PI3K, p38 and MAPK pathways are involved in BMP‐2‐induced mononuclear cell migration

To characterize the signalling cascades activated by BMP‐2 in monocytes, we stimulated monocytes with BMP‐2 and analysed the activation of different pathways by Western blot analysis. BMP‐2 induced the phosphorylation of Smad1 (Figure 1C), as previously reported and in addition the phosphorylation of AKT, p38 and ERK (Figure 1C).

To determine the functional role of PI3K, p38 and ERK pathways in BMP‐2‐induced monocyte chemotaxis, we made use of chemical kinase inhibitors. Pharmacological inhibition of PI3K abrogated basal motility of monocytes and diminished BMP‐2‐induced chemotaxis (Figure 1D). Treatment of monocytes with a p38 kinase inhibitor prior to the chemotaxis assay inhibited both basal and BMP‐2‐induced monocyte chemotaxis (Figure 1D). Finally, although inhibition of the ERK1/2 pathway did not affect basal monocyte motility, it interfered with BMP‐2‐induced monocyte migration (Figure 1E).

### 3.3 T2DM induces BMP‐2 expression but it does not affect BMP‐2‐induced monocyte migration

**Figure 2:**
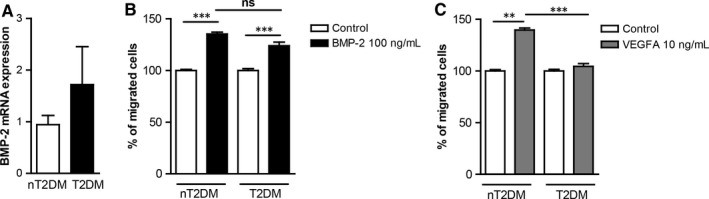
T2DM interferes with VEGFA‐induced, but not with BMP‐2‐induced monocyte migratory responses and enhances BMP‐2 gene expression in monocytes. A. Monocytes were isolated from the blood of patients with T2DM (n = 10) or without T2DM (nT2DM, n = 20), and the mRNA expression of BMP‐2 was analysed by qRT‐PCR. B, C. Monocytes were isolated from patients with T2DM (n = 12) or without T2DM (nT2DM, n = 20). The monocyte chemotactic responses towards BMP‐2 (B) and VEGFA (C) were analysed. Data are represented as mean ± SEM. (**P < 0.01, ***P < 0.001)

Increased BMP‐2 levels are associated with atherosclerosis in T2DM patients.^15^ T2DM is a cardiovascular risk factor, which results in monocyte dysfunction^3^, ^4^). We have analysed BMP‐2 mRNA expression in monocytes from non‐T2DM (nT2DM) and T2DM patients. Our results show that BMP‐2 gene expression is increased in monocytes from T2DM individuals (Figure 2A). To characterize the effects of T2DM on BMP‐2‐induced monocyte migration, monocytes from patients with and without T2DM were analysed for their migratory responses towards BMP‐2 and VEGFA. Monocytes from nT2DM patients migrate towards both, BMP‐2 and VEGFA. However, although monocytes from T2DM patients do not respond to VEGFA‐induced migration, they can still respond to BMP‐2 (Figure 2B and C).

### 3.4 BMP‐2 hinders monocyte differentiation into M2 macrophages

**Figure 3:**
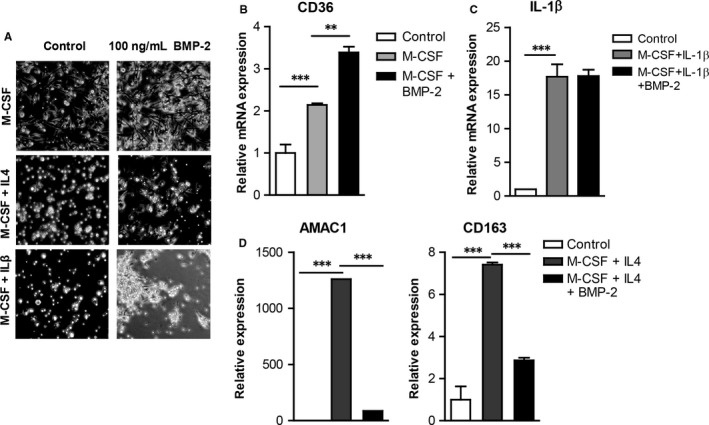
BMP‐2 enhances the expression of macrophage markers, but interferes with the expression of M2 macrophages markers. A, B, Monocyte differentiation into macrophages was induced by addition of 50 ng/mL M‐CSF in the presence or absence of BMP‐2. Differentiation into M1 and M2 macrophages was induced with 10 ng/mL IL‐1β or 10 ng/ml IL‐4, respectively, in the presence or absence of BMP‐2. A, Morphology of monocyte‐derived M0, M1 and M2 macrophages was revealed by phase contrast microscopy. B‐C, The expression of the CD36 macrophage marker, M1 markers Il‐1β and M2 macrophage markers AMAC1 and CD163 was analysed by qRT‐PCR (n = 5). Data are represented as mean ± SEM. (**P < 0.01, ***P < 0.001)

After entering a tissue monocytes differentiate into macrophages.^23^ M1 macrophages promote inflammatory responses, while M2 macrophages are anti‐inflammatory and play an important role in resolution of inflammation, tissue repair and wound healing.^24^ To characterize the effects of BMP‐2 on monocyte differentiation into macrophages, monocytes were differentiated into macrophages and then in M1 or M2 macrophages in the presence or absence of BMP‐2 (Figure 3A). BMP‐2 enhanced expression of the macrophage marker CD36 by 1.7‐fold (Figure 3B). However, BMP‐2 interfered with monocyte differentiation into M2 macrophages as it attenuated expression of AMAC1 and CD163 by 15‐ and 2.5‐fold, respectively (Figure 3D). BMP‐2 had no effects on the expression of IL‐1β, an M1 macrophage marker (Figure 3C).

### 3.5 BMP‐2 induces mononuclear cell adhesion

**Figure 4:**
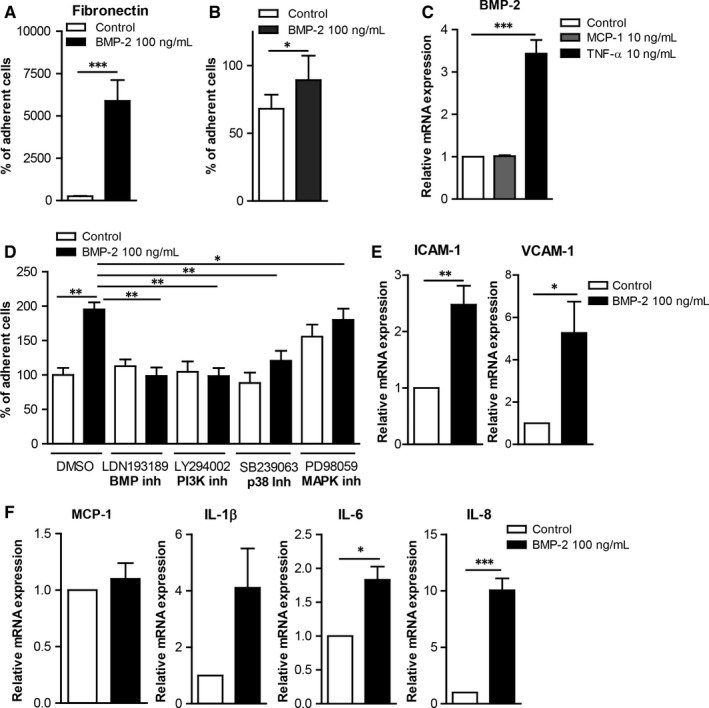
BMP‐2 induces mononuclear cell adhesiveness in human endothelial cells (ECs). A, Monocytes were incubated for 30 min with or without 100 ng/mL BMP‐2 and allowed to adhere on Fibronectin‐coated plates for 30 min, and the number of adherent cells was counted (n = 5). B, HUVECs were stimulated with 10 ng/mL Tumour necrosis factor‐α (TNF‐α) or 10 ng/mL MCP‐1 for 5 h. The mRNA expression of BMP‐2 was analysed by qRT‐PCR (n = 5). C, Freshly isolated monocytes were stimulated for 30 min with 100 ng/mL BMP‐2, labelled with Calcein and allowed to adhere on HUVECs, adherent cells were quantified (n = 5). D, HUVECs were preincubated with vehicle, 3 μmol/L LDN193189 BMP receptor kinase inhibitor, 1 μmol/L LY29400 PI3K inhibitor, 10 μmol/L SB239063 p38 kinase inhibitor or 25 μmol/L PD98059 MAPK inhibitor and then stimulated with 100 ng/mL BMP‐2 for 5 h. Labelled monocytes were allowed to adhere on them for 20 min, and adherent cells were quantified (n = 5). E‐F, HUVECs were stimulated with 100 ng/mL BMP‐2 for 5 h. The mRNA expression of ICAM‐1, VCAM‐1, MCP‐1, IL‐1β, IL‐6 and IL‐8 was analysed by qRT‐PCR (n = 6). Data are represented as mean ± SEM. (*P < 0.05, **P < 0.01, ***P < 0.001)

Monocyte adhesion on extracellular matrix and on ECs plays a crucial role in the development of atherosclerosis, thus we investigated whether BMP‐2 affects monocyte adhesion. Interestingly monocyte stimulation with BMP‐2 induced their adhesion to fibronectin by 6000‐fold (Figure 4A).

Tumour necrosis factor (TNF)‐α induces inflammatory responses in monocytes as well as in ECs. Our gene expression analysis results revealed that TNF‐α induced BMP‐2 mRNA expression in HUVECs as well as in primary monocytes (Figure 4B and Figure S1), suggesting an important role of BMP‐2 in EC inflammation. However, MCP‐1 did not induce BMP‐2 expression in HUVECs or in primary monocytes. To analyse the effects of BMP‐2 on the interaction of mononuclear cells with endothelial cells, we analysed the effect of BMP‐2 on mononuclear cell adhesiveness. BMP‐2 stimulation of monocytes induced their adhesiveness on HUVECs (Figure 4C).

**Figure 5:**
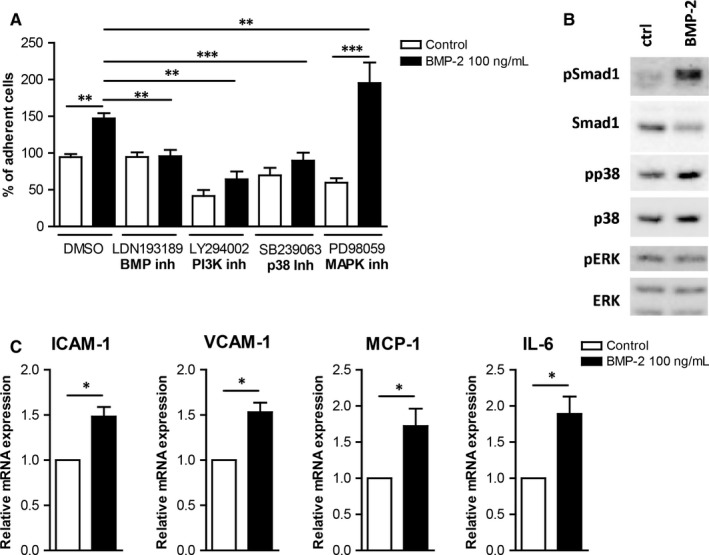
BMP‐2 induces endothelial cell adhesiveness in mouse endothelial cells (ECs). A, bEnd5 cells were preincubated with vehicle, 3 μmol/L LDN193189 BMP receptor kinase inhibitor, 1 μmol/L LY29400 PI3K inhibitor, 10 μmol/L SB239063 p38 kinase inhibitor or 25 μmol/L PD98059 MAPK inhibitor and then stimulated with 100 ng/mL BMP‐2. Calcein‐labelled primary monocytes were allowed to adhere on them for 20 min. Nonadherent cells were washed, and adherent cells were quantified using ImageJ (n = 5). B, bEnd5 cells were stimulated with 100 ng/mL BMP‐2 for 20 min. Protein lysates were analysed by Western blot for phosphorylation of downstream signalling regulators. Smad1, p38, ERK1/2. C, bEnd5 cells were stimulated with 100 ng/mL BMP‐2 for 5 h. The mRNA expression of ICAM‐1, VCAM‐1, MCP‐1 and IL‐6 was analysed by qRT‐PCR (n = 6). Data are represented as mean ± SEM. (*P < 0.05, **P < 0.01, ***P < 0.001)

In addition, BMP‐2 stimulation of HUVECs or the mouse endothelial cell line bEnd5 increased their adhesiveness to monocytes (Figure 4D and Figure 5A). To characterize the mechanisms mediating the BMP‐2 effects on EC adhesiveness, we analysed the expression of adhesion and inflammatory molecules. BMP‐2 significantly induced the expression of ICAM‐1 and VCAM‐1 by 2.5‐ and 5‐fold, respectively (Figure 4E). BMP‐2 also induced the expression of the pro‐inflammatory cytokines IL‐1β, IL‐6 and IL‐8 by 4‐, 1.8‐ and 10‐fold, respectively (Figure 4F) Similarly BMP‐2 induced the expression of ICAM‐1, VCAM‐1, MCP‐1 and IL‐6 in bEnd5 ECs (Figure 5C).

The signalling cascades activated by BMP‐2 in ECs were analysed to characterize the molecular mechanisms by which BMP‐2 induces ECs adhesiveness. BMP‐2 stimulation of mouse bEnd5 resulted in phosphorylation of Smad1/5 and p38 (Figure 5B). BMP‐2 stimulation did not affect the ERK1/2 pathway. To characterize the functional role of these pathways on BMP‐2‐induced EC adhesiveness, we used kinase inhibitors against the BMP, p38, ERK/MEK and PI3K signalling cascades. Inhibition of BMP, PI3K and p38 pathways resulted in reduced basal and BMP‐2‐induced monocyte adhesion on both HUVECs and bEnd5 (Figure 4D and 5A). In contrast, although inhibition of ERK1/2 signalling inhibited BMP‐2‐induced monocyte adhesion on HUVECs, it resulted in increased levels of mononuclear cell adhesion on bEnd5 cells (Figure 5A).

## 4 DISCUSSION

BMP cytokines play important roles in vascular development and deregulation of BMP pathways lead to vascular abnormalities and cardiovascular diseases. Several studies have suggested an important role for BMP‐2 in endothelial cell inflammation, vascular calcification and development of atherosclerosis.^9^, ^10^, ^11^, ^18^, ^25^ Although the effects of BMP‐2 on EC and SMC have been studied extensively, their effects on mononuclear cell function have not been investigated yet.

The results of the current study demonstrate that BMP‐2 can induce monocyte migration in a concentration‐dependent manner. Inhibition of endogenous BMP‐2/4/7 signalling, using the natural antagonist Noggin and the BMP receptor kinase inhibitor LDN193189, resulted in reduced monocyte chemokinesis, as well as of MCP‐1‐induced chemotaxis. In addition, our results suggest that MCP‐1 does not induce expression of BMP‐2 in primary human monocytes. Taken together, our results suggest that endogenous BMP‐2 signalling plays a crucial role in mononuclear cell motility. Hence, BMP‐2 may contribute to the development of atherosclerosis by inducing the infiltration of mononuclear cells in the vessel wall. Our data show that BMP‐2 induces monocyte migration via PI3K and MAPK pathways. These results are in line with previous results showing, that PI3K and MAPK activity play crucial roles in both basal monocyte motility and growth factor‐induced monocyte chemotaxis.^4^, ^19^ In line with our results, previous studies have suggested that BMP‐2 can induce migration of endothelial cells *in vitro* and *in vivo*,^26^, ^27^ as well as of bone marrow mesenchymal progenitors and osteoblasts.^28^, ^29^

Recent studies suggested that patients with T2DM have higher circulating levels of BMP‐2 than normal controls and that increased plasma BMP‐2 levels are associated with atherosclerosis and coronary calcification in T2DM patients.^15^ Several studies have indicated that sex hormones alter the immune responses during atherosclerosis, resulting in different disease phenotypes. Nevertheless, there is no evidence that there are significant differences in BMP‐2 expression levels between females and males in the context of T2DM.^15^, ^30^, ^31^ This might be due to the low number of individuals analysed per study. Future studies in a larger cohort of patients adjusted for the sex, age and other factors might provide further information on whether BMP‐2 levels are different in females and males in T2DM. In addition, it was also shown that Noggin expression reduces glycaemia and vascular inflammation in *db/db* mice^32^ and it was suggested that T2DM induces vascular inflammation by altering the balance between BMP‐2/4 and noggin.^11^ In line with this, we demonstrate that monocytes from T2DM patients express higher levels of BMP‐2 mRNA further supporting the notion that T2DM results in increased expression of BMP‐2. Circulating monocytes are recruited to sites of arteriogenesis by MCP‐1, but also VEGFA and contribute to formation of new collaterals.^3^, ^33^ T2DM results in mononuclear cell dysfunction and impedes VEGFA‐induced mononuclear cell responses, which has been suggested to lead to the decreased formation of collateral vessels, seen in patients with T2DM.^34^, ^35^ Conversely, it was shown that increased monocyte accumulation contributes to the development of atherosclerosis.^1^ We demonstrate that although monocytes from T2DM patients display attenuated chemotactic responses towards VEGFA, they still respond to BMP‐2 induced migration. In addition, we demonstrate that TNF‐α induces the expression of BMP‐2 in HUVECs, suggesting a pro‐inflammatory role for BMP‐2. These data suggest that in T2DM patients, BMP‐2 can promote atherosclerosis development by inducing monocyte accumulation to sites of inflammation.

While BMP‐2 potentiates monocyte differentiation into macrophages, it interferes with monocyte differentiation into M2 macrophages, as elucidated in the current study. Atherosclerotic plaque progression is associated with an increase in M1 pro‐inflammatory macrophages compared to the number of anti‐inflammatory M2 macrophages.^36^ Our results suggest that BMP‐2 promotes inflammatory responses by interfering with the resolution of inflammatory responses as it obstructs the differentiation of macrophages into the M2 anti‐inflammatory macrophages and this way contribute to the development of atherosclerosis. In line with this, it has been shown that human monocytes and macrophages undergo M1‐like inflammatory polarization when exposed to high levels of glucose on in vitro culture conditions and in patients with hyperglycaemia, suggesting that increased levels of BMP‐2 in T2DM patients may also contribute to the enhancement of inflammatory responses.^37^, ^38^, ^39^, ^40^

The interaction between mononuclear cells and vascular wall facilitates their migration into the plaque microenvironment and the development of atherosclerosis.^1^ BMP‐2 signalling induces mononuclear cell adhesiveness on fibronectin and on ECs. In addition, we demonstrate that BMP‐2 induces inflammatory responses in human and mouse ECs and enhances their adhesiveness to mononuclear cells. Our results are in line with a previous study that demonstrated that BMP‐2 induces adhesiveness of HCAECs.^41^ We now demonstrate that several signalling cascades such as BMP, PI3K, p38 and ERK are involved in BMP‐2‐induced EC adhesiveness. Although inhibition of the ERK signalling cascade resulted in inhibition of BMP‐2‐induced adhesiveness in HUVECs (Figure 4D) and in HCAECs^41^, it potentiated BMP‐2‐induced bEnd5 adhesiveness. This discrepancy is probably due to the context‐dependent effects of BMP ligands as it has been reported before for several members of the TGF‐β superfamily.^6^ BMP‐2‐induced EC adhesiveness is probably due to BMP‐2‐induced expression of adhesion molecules, as well as pro‐inflammatory cytokines on ECs. Our results suggest that BMP‐2, by increasing adhesion of monocytes on ECs, contributes to the increased inflammatory responses during atherosclerosis.

Our results provide important insights into the molecular mechanism of BMP‐2‐mediated signalling in monocytes and their interaction with ECs. We demonstrate that BMP‐2 may exert its pro‐inflammatory function in atherosclerosis by endorsing monocyte recruitment, their adhesion to the endothelium and by interfering with monocyte‐to‐macrophage differentiation into the anti‐inflammatory M2 macrophages. Thus, BMP‐2 may be a therapeutic target for prevention of atherosclerosis.

## CONFLICT OF INTERESTS

The authors confirm that there are no conflict of interests.
