# Supplementary figures and images for: BMP‐2 induces human mononuclear cell chemotaxis and adhesion and modulates monocyte‐to‐macrophage differentiation
Source: J Cell Mol Med. 2018 Aug 13;22(11):5429–38. doi: 10.1111/jcmm.13814 (PMC6201342; doi:10.1111/jcmm.13814)

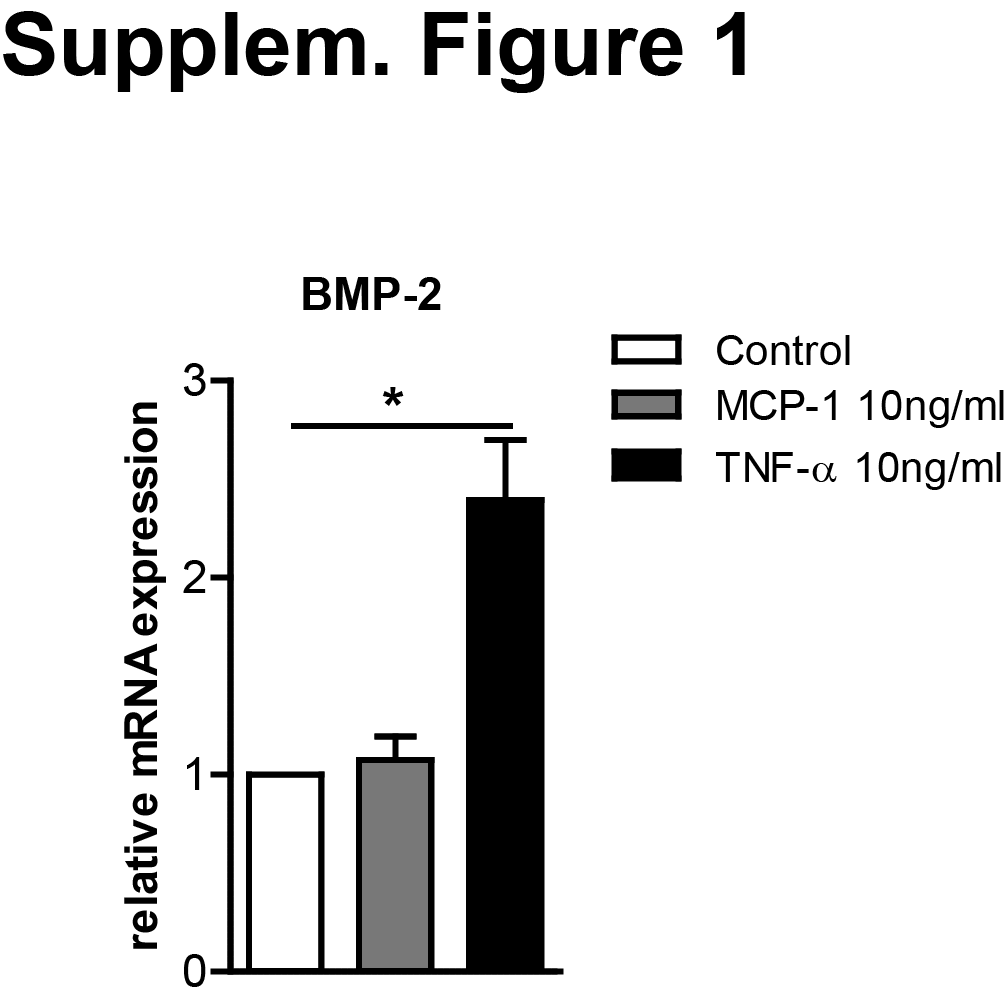

Supplement: Supplementary file 1 [file JCMM-22-5429-s001.tif]
