# Supplementary material for: BMP‐2 induces human mononuclear cell chemotaxis and adhesion and modulates monocyte‐to‐macrophage differentiation
Source: J Cell Mol Med. 2018 Aug 13;22(11):5429–38. doi: 10.1111/jcmm.13814 (PMC6201342; doi:10.1111/jcmm.13814)
Supplement: Supplementary file 2 [file JCMM-22-5429-s002.docx]

**Supplementary information**

BMP-2 induces human mononuclear cell chemotaxis and adhesion and modulates monocyte-to-macrophage differentiation

**Evangelia Pardali^1,2*^, Lena-Maria Makowski^1,2^, Merle Leffers^1,2^, Andreas Borgscheiper^1,2^ and Johannes Waltenberger^1,2*^**

1 Department of Cardiovascular Medicine, University Hospital of Münster, Albert-Schweitzer- Campus 1, 48149 Münster, Germany

2 Cells-in-Motion Cluster of Excellence (EXC 1003 - CiM), University of Münster, 48149 Münster, Germany

**Supplementary Table 1: Primer sequences for human genes used for real-time RT-qPCR.**

| **Name** | **Sequence** |
| --- | --- |
| hGAPDH-F | 5’-CAAGGCTGTGGGCAAGGT-3’ |
| hGAPDH-R | 5’-GGAAGGCCATGCCAGTGA-3’ |
| h18S rRNA-F | 5’-CGGCGACGACCCATTCGAAC-3’ |
| h18S rRNA-R | 5’-GAATCGAACCCTGATTCCCCGTC-3’ |
| hBMP-2-F | 5’-TTAAGTTCTATCCCCACGGAGG-3’ |
| hBMP-2-R | 5’-CCCACCTGCTTGCATTCTGATT-3’ |
| hMR-F | 5’-CGAGGAAGAGGTTCGGTTCACC-3’ |
| hMR-R | 5’-GCAATCCCGGTTCTCATGGC-3’ |
| hCD36-F | 5’-TCAGCAAATGCAAAGAAGGGAGAC-3’ |
| hCD36-R | 5’-GGTTGACCTGCAGCCGTTTTG-3’ |
| hAMAC1-F | 5’-AGCTCTGCTGCCTCGTCTAT-3’ |
| hAMAC1-R | 5’-CCCACTTCTTATTGGGGTCA-3’ |
| hICAM1-F | 5’-GCTTCGTGTCCTGTATGGC-3’ |
| hICAM1-R | 5’-CTGGCGTTATAGAGGTACG-3’ |
| hVCAM1-F | 5’-TTCTGAGAGTGTCAAAGAAGG-3’ |
| hVCAM1-R | 5’-AAGGAGGATGCAAAATAGAGC-3’ |
| hIL-1β-F | 5’-GGGCCTCAAGGAAAAGAATC-3’ |
| hIL-1β-R | 5’-TTCTGCTTGAGAGGTGCTGA-3’ |
| hIL-6-F | 5’-AGTGCCTCT TTGCTGCTTTCAC-3’ |
| hIL-6-R | 5’-TGACAAACAAATTCGGTACATCCT-3’ |
| hIL-8-F | 5’-ACTGAGAGTGATTGAGAGTGGAC-3’ |
| hIL-8-R | 5’-AACCCTCTGCACCCAGTTTTC-3’ |

**Supplementary Table 2: Primer sequences for mouse genes used for real-time RT-qPCR.**

| **Name** | **Sequence** |
| --- | --- |
| mGAPDH-F | 5’-AACTTTGGCATTGTGGAAGG-3’ |
| mGAPDH-R | 5’-ACACATTGGGGGTAGGAACA-3’ |
| mICAM1-F | 5’-GGCATTGTTCTCTAATGTCTCCG-3’ |
| mICAM1-R | 5’-GCTCCAGGTATATCCGAGCTTC-3 |
| mVCAM1-F | 5’-AGTTGGGGATTCGGTTGTTCT-3’ |
| mVCAM1-R | 5’-CCCCTCATTCCTTACCACCC-3’ |
| mMCP-1-F | 5’-CCAGAGCCAACGTCAAGCAT-3’ |
| mMCP-1-R | 5’-CAGCCGTGCAACAATCTGAA-3’ |
| mIL-6-F | 5’-GAGGATACCACTCCCAACAGACC-3’ |
| m IL-6-R | 5’-AAGTGCATCATCGTTGTTCATACA-3’ |

**Supplementary Figure legends**

**Supplementary Figure 1** Effects of MCP-1 and TNF-α on BMP-2 expression in primary monocytes. Human primary were stimulated with 10 ng/ml TNF-α or 10ng/ml MCP-1 for 6 hours. The mRNA expression of BMP-2 was analyzed by qRT-PCR (n=5). Data are represented as mean ± SEM. (*p< 0.05).
